# Prevalence, Etiology, and Clinical Outcomes of Healthcare-Associated Infections in a Tertiary Pediatric Hospital

**DOI:** 10.3205/dgkh000677

**Published:** 2026-09-10

**Authors:** Shahnaz Armin, Rahele Razmara, Sepideh Mirzayi, Leila Azimi, Abdollah Karimi

**Affiliations:** 1 Pediatric Infections Research Center, Research Institute for Children’s Health, Shahid Beheshti University of Medical Sciences, Tehran, Iran; 2 Mofid children hospital, Shahid Beheshti University of Medical Sciences, Tehran, Iran

**Keywords:** health association infections, mortality, hospital stay, antibiotic resistance

## Abstract

**Background:**

Healthcare-associated infections (HAIs) are becoming an ever more prominent concern within hospitals and global challenges which can increase the rate of mortality and morbidity, especially in children. This study aimed to survey the prevalence of HAIs in a referral children’s hospital in Iran, including their frequency, isolated bacteria, antibiotic resistance patterns, mortality rates, and length of hospital stay.

**Method:**

In this cross-sectional study, all pediatric patients hospitalized for more than 48 hours were surveyed, and patients confirmed with HAI were included. The information of patients was extracted from their records.

**Results:**

The prevalence of HAIs was 5%. Patients with HAIs experienced a significant increase in both mortality rates and length of hospitalization. The study also highlighted the prevalence of high-carbapenem-resistant strains of *Pseudomonas aeruginosa* and *Acinetobacter baumannii *as major contributors to HAIs.

**Conclusion:**

This study highlights the significance of HAIs and their impact on patient mortality, as well as the duration of hospital stays. Furthermore, it is important to consider the antibiotic resistance of the causative pathogens.

## Zusammenfassung

**Hintergrund:**

Healthcare-assoziierte Infektionen (HAIs) werden in Krankenhäusern zu einem immer größeren Problem und stellen eine globale Herausforderung dar, die insbesondere bei Kindern zu einem Anstieg der Mortalität und Morbidität führen können. Ziel der Studie war es, die Prävalenz von HAIs, ihre Ätiologie Erregeresistenz, Mortalität und Einfluss auf die Dauer des Krankenhausaufenthalts in einer Kinderklinik im Iran zu untersuchen.

**Methode:**

In dieser Querschnittsstudie wurden alle pädiatrischen Patienten erfasst, die länger als 48 h stationär behandelt wurden, wobei nur Patienten berücksichtigt wurden, bei denen eine HAI bestätigt worden war. Die Patientendaten wurden den Krankenakten entnommen.

**Ergebnisse:**

Die Prävalenz der HAIs lag bei 5%. Bei Patienten mit einer HAI kam es zu einem signifikanten Anstieg sowohl der Mortalität als auch der Dauer des Krankenhausaufenthalts. Als Erreger standen resistente Carbapenem-Stämme von *Pseudomonas aeruginosa* und *Acinetobacter baumannii* im Vordergrund.

**Schlussfolgerung:**

Die Studie verdeutlicht die Bedeutung von HAIs und deren Auswirkungen auf die Mortalität sowie auf die Dauer des Krankenhausaufenthalts. Darüber hinaus ist es wichtig, die Antibiotikaresistenz der verursachenden Erreger zu berücksichtigen.

## Introduction

Health association infections (HAIs) are recognized as a common complication among hospitalized patients. These infections can lead to elevated patient morbidity, hinder the effectiveness of the treatment for the primary illness, prolong hospital stays, thereby incurring additional costs for the healthcare system, and may finally result in patient mortality, especially among children and neonates [1], [2].

Establishing and executing effective infection control programs to control the transmission of antimicrobial resistant (AMR) bacteria within clinics and hospitals has proven essential in decreasing infection rates and plays a crucial role [1]. AMR has quickly become a major worldwide issue in the 21^st^ century [3], [4]. AMR ranks as the third leading global cause of mortality and it has appeared as one of the important global public health threats [3], [4]. Recent data reveals that approximately 4.95 million deaths occurred in 2019 as a result of bacterial resistance to antibiotics. AMR, often named the “Silent Pandemic” presents a pressing issue that demands immediate attention and more efficient management. Predictions suggest that the number of fatalities due to antibiotic resistance is projected to climb significantly and it could potentially become the world’s primary cause of death by the year 2050. Moreover. Also, 90% of infants diagnosed with sepsis and requiring intensive care unit (ICU) admission in the Middle Eastern region were found to have been infected with resistant bacteria [3], [4], [5], [6], [7]. The Word Health Organization (WHO) and various other organizations and researchers are in consensus on the pressing need for a worldwide, collaborative strategy to combat the alarming spread of AMR [4], [7], [8], [9]. It is crucial to consider the data on antibiotic resistance patterns, particularly in HAIs. It can lead cause to higher rates of death and illness in the Pediatric Intensive Care Unit (PICU). This can lead to an increase in the risk of death, the level of sickness, the time spent in the hospital, and the overall cost of treatment which including heightened medication consumption and diagnostic tests [10], [11]. As per a recent report released by the WHO regarding 55 hospitals across 14 different countries, it was found that 8.7% of patients admitted had HAIs. this study indicated that HAIs were more commonly observed in the Eastern Mediterranean Region compared to the Western Pacific Region [11], [12], [13]. We design this study to determine the antibiotic resistance patterns of isolated bacteria from clinical samples in patients with HAIs in Mofid Children Hospital.

## Method

### Study design and setting

A hospital-based cross-sectional study was conducted from May 21, 2023, to August 21, 2023, at Mofid Children’s Hospital, Iran (the age all of the patients under 18 years old). Mofid Referral children Hospital is a referral and teaching hospital in Tehran, Iran. According to annual report of the 2024 admission rate was over 21,600 patients. The hospital has a total of 295 inpatient beds.

#### Study population and inclusion criteria

Every patient admitted to the hospital was included in the study, with only those who remained at least 48 hours being considered. Patients who exhibited signs and symptoms of infection within the initial 48 hours were not included in the study. 

#### Data collection

During the study period, all children who developed signs and symptoms of infection after 48 hours of hospitalization which identified by the infection control nurse in various hospital departments included in the study as HAIs patients. Their demographic information was collected by the same nurse. 

#### Operational definition

HAIs refer to infections that are either localized or systemic and are caused by an adverse reaction to an infectious agent or its toxin. These infections typically occur 48 hours or more after a patient has been admitted to a hospital while receiving medical treatment in a healthcare and were not present at the time of admission [14]. The HAIs which our considered were; bloodstream infections (BSI), urinary tract infections (UTI), ventilator-associated pneumonia (VAP), pneumonia (PNEU), surgical site infections (SSI), gastrointestinal system infection (GI), lower respiratory system infection (LRI), based on national Iran health ministry guideline [15].

In our setting, the HAIs were divided to two categories: Clinical HAIs confirmed. Laboratory HAIs confirmed. In pursuit of our objective, we directed our efforts towards analyzing laboratory-confirmed HAIs for this study.

#### Bacterial diagnostic and antibiotic susceptibility testing

The bacteria that cause of HAIs were isolated from blood, urine, cerebrospinal fluid (CSF) and other localization. Bacteria were identified using specialized biochemical and microbiological techniques. The antibiotic susceptibility testing was prepared by disc diffusion method against selected antibiotics according to the guideline 2023 of the Clinical and Laboratory Standards Institute. Antibiotics include ampicillin (10 µg), ampicillin/sulbactam (10/10 µg), aztreonam (30 µg), amoxicillin/clavulanic acid (20/10 µg), amikacin (30 µg), imipenem (10 µg), piperacillin (100 µg), piperacillin/tazobactam (100/10 µg), tetracycline (30 µg), tobramycin (10 µg), gentamicin (10 µg), cefazolin (30 µg), cefepime (30 µg), ceftazidime (30 µg), ceftriaxone (30 µg), cefotaxime (30 µg), cefuroxime (30 µg), ciprofloxacin (5 µg), cotrimoxazole (1.25/23.75 µg), meropenem (10 µg), vancomycin (30 µg), and linezolid (30 µg).

#### Data analysis

Data were entered into SPSS V.23 statistical software for further analysis. Descriptive statistics were computed to present the frequency distribution of important variables. The cumulative prevelance proportion was calculated as the number of new HAI cases per person in the population over a defined period of time; and it is the probability of developing HAIs over a stated study period (3 months). We estimated the prevalence rate as the number of HAI cases per unit of time, and the denominator represents the total amount of time ‘at-risk’ without experiencing HAIs for all children whom were being followed for 3 months. 

#### Ethics

The study was conducted in accordance with the Declaration of Helsinki, and approved by the Ethics Committee of the Research Institute for Children’s Health, Shahid Beheshti University of Medical Sciences (IR.SBMU.RICH.REC.1403.002). All participants or from their parents or legal guardians (for individuals younger than the age of 16) signature the consent form. 

## Results

Over the period of three months, a total of 5,400 hospitalized patients underwent evaluation and among them, 3,928 individuals (73%) who remained hospitalized for over 48 hours participated in the study. All 3,928 patients were monitored throughout their stay in our hospital, with follow-ups continuing until they were either discharged, transferred to another facility, or passed away. 203 (5%) of them showed signs and symptoms of infections and enrolled in this study as HAIs patients (Figure 1 (Fig. 1)). The highest prevalence of HAIs was observed in the Pediatric Intensive Care Unit (PICU). 102 (50.2%) of children with HAIs were female and 101 (49.8%) were male.

Among 203 cases, 139 (68%) were verified through laboratory testing, which detected the presence of bacteria and fungi, while the remaining 65 (32%) were identified as clinical HAIs (Figure 1 (Fig. 1)). The incidence rate of HAIs was 5,168 per 100,000 patients (5.1%).

The mean hospital stays of the patients who stayed more than 48 hours without HAIs was 5.97±0.2 days and for patients with HAIs 26.3±22.5 days. The mean days of hospitalization up to observed HAIs were 13.2±14.7 days. 

The mortality rate was 1% and 18% among patients with HAIs and without HAIs, retrospectively.

The hospital stay and mortality rate of the patients with HAIs was statistically significant (P value <0.0001) compared to patients without HAIs.

Table 1 (Tab. 1) illustrates the prevalence rates of different types of HAIs, with laboratory-confirmed BSI being the most frequently reported and the most common type of HAIs. 

The patients with VAP had the highest mortality rate among all HAIs at 44%, with laboratory confirmed BSI following behind at 22%.

*Pseudomonas (P.) aeruginosa* was the most isolated microorganisms with 29% frequency among laboratory confirmed HAIs. The other isolated pathogens show in Table 2 (Tab. 2). All identified bacteria caused the most of the BSI in HAIs just *Escherichia (E.) coli*. 

The antibiotic susceptibility results of Gram-negative isolated bacteria are shown in Table 3 (Tab. 3). 

In *P. aeruginosa*, Enterobacter spp., *K. pneumoniae*, *A. baumannii* and *E. coli* the most of resistant was observed against, aztreonam, ampicillin, ampicillin, (meropenem and cefepime) and cefotaxime, respectively. The highest resistance rate in Enterococcus spp. was recorded against aztreonam at 92%, followed by vancomycin at 67% and ampicillin at 60%. All strains exhibited susceptibility to linezolid.

## Discussion

The results indicated that the prevalence of HAIs in our hospital was 5%, with the majority observed in the PICU. In 2019 a study carried out in Iran, the average prevalence of HAIs in pediatric wards was reported to be 7.8%, which is higher than in our study. This discrepancy may be attributed to the differences in location and healthcare facilities [16]. But similar to our results in a study in Turkey, 2021, the rate of HAIs was 5.6% [17]. The date from Iranian nosocomial infection surveillance in 2020 reported an overall rate of HAIs at 26.57 per 1,000 patients, which corresponds to a prevalence of 2.62% [18]. This result is lower than ours, likely due to the fact that our survey was conducted in specialized pediatric hospitals that have cancer and transplant units, as well as a PICU, where the utilization of invasive medical devices is common. The study conducted by the CDC indicated that the rate of HAIs ranged from 12.2 to 14.9%, or from 5.8 to 19.0 infections per 1,000 patient days [19]. The variations may depend on factors such as the age of the population, the prevalence of specific diseases, hospital capacity, and compliance with infection prevention guidelines. Based on the random-effects model, the rate of HAIs in a Middle Eastern country from 1995 to 2020 indicated that the overall prevalence of HAIs in Iran was 4.5% [95% CI: 3.5 to 5.7] [20]. In 2018, a study across 940 hospitals in Iran, revealing a national average of the incidence rate was 4.2 per 1,000 patient-days [21]. Our results surpassed those of the previously mentioned studies [20], [22], likely attributable to differences in the population and the criteria used for the identification of HAIs in each instance. 

The high rate of isolated Gram-negative bacteria, especially *P. aeruginosa* and a study in Egypt [22] from patients with HAIs has two clinical significances: A high prevalence of antibiotic-resistant strains, mainly beta-lactam resistant, combined with limited therapeutic options and a higher associated mortality in pediatric with HAIs. 

The significantly elevated mortality rate and average length of hospitalization (P<0.0001) in patients with HAIs compared to those without HAIs in our study underscores the critical importance of HAIs and the necessity of prevent them. This concern was also noted in a study conducted in Iran in 2018 [23]. The average duration of hospitalization for patients with HAIs in this study was 26.3±22.5 days, which is similar to a study conducted in Iran in 2020, where the mean length was reported as 29.22±45.1 days [18]. 

BSI was the most frequently observed HAI in our study like a study in Turkey in 2021 [17]; however, the majority of mortality was associated with VAP in our study. In a multicenter study conducted in Iran in 2017, VAP was identified as the predominant type of HAI [18]. The variation may stem from the distinct populations selected for each study. In the present research, the focus was solely on pediatric patients, while the study conducted by Izadi et al. [18] included participants from both pediatric and adult age groups.

A meta-analysis conducted in 2018, which examined research from Iran, indicated that BSIs were associated with the highest rates of HAIs [20]. Furthermore, other studies, including a Turkish study conducted in 2021 and an Indian study, confirmed this [17], [18], [24], [25]. 

HAIs were found to be more common in ICU settings in this study. Identical results have been reported in various other research studies [18], [26], [27].

This investigation identified *P. aeruginosa* as the most common isolate in HAIs, followed by *K. pneumoniae*. However, the dominant pathogens can vary geographically and temporally, as demonstrated by a 2019 study in Urmia, Iran, which found *E. coli* and *A. baumannii* to be prevalent [28] and a Turkish study that identified *A. baumannii* as the primary cause of HAIs [29]. The discrepancies can be ascribed to the varying cities, specific years of research, and the selected populations. Nevertheless, all these types of bacteria are widely recognized as causative agents of HAIs.

*E. coli* and *K. pneumonia* infections emerged as the most frequently reported causes of HAIs in a systematic literature review and meta-analysis conducted in a Middle Eastern country from 1995 to 2020 [20]. *Klebsiella* spp. infection is the second cause of HAIs in the current study and this review [20]. 

The average resistance rate to aminoglycosides is 48.6% in *P. aeruginosa* strains in the recent study. This is considerably lower than the Tehran study’s finding of 64.8%, which indicates a higher prevalence of resistance in that population [30]. The unique characteristics of our patient group may have influenced the observed resistance patterns, emphasizing the importance of context when interpreting antibiotic resistance data [30]. These contrasting results likely stem from various factors, such as differences in patient demographics and selection criteria, particularly regarding the pediatric cohort utilized in our study. Also, the rate of antibiotic resistance is expected to be lower in bacteria isolated from infections in children due to their lower exposure to antibiotics than in adults.

Our study revealed no instances of carbapenem-resistant *E. coli*, a notable finding given that a Tehran study conducted between March 2017 and March 2018 reported a prevalence of 2.3% for this specific antibiotic resistance [30]. In contrast, a Turkish study on adult from 2023 indicated carbapenem resistance rates of 90.4% for *A. baumannii* and 48.8% for *P. aeruginosa* [29]. These discrepancies may stem from differences in the study methodologies, focus areas and different treatment guidelines and antibiotic stewardship in different country.

## Limitations

In this study, HAIs could not be followed up after the patients were discharged, except for cases where the patient returned to the hospital.

## Conclusion

The significant difference in the increase in mortality and the length of hospital stay in children with HAIs demonstrate the importance of implementation of continuous surveillance and intervention programs. Moreover, the high frequency of carbapenem-resistant *A. baumannii* and *P. aeruginosa* was alarming for the nosocomial infection committee in our hospital to introduce infection control protocols. 

## Notes

### Authors’ ORCIDs

- Armin S: https://orcid.org/0000-0002-4993-482X
- Razmara R: https://orcid.org/0000-0002-0858-7331
- Mirzayi S: https://orcid.org/0009-0008-0202-8804
- Azimi L: https://orcid.org/0000-0002-7216-2530
- Karimi A: https://orcid.org/0000-0002-4225-0097

### Ethical approval

The study was approved by the Ethics Committee of the Research Institute for Children’s Health, Shahid Beheshti University of Medical Sciences.

### Funding

This study was conducted with the financial support of Pediatric Infections Research Center (Grant No. 43005935), Shahid Beheshti University of Medical Sciences, Tehran, Iran.

### Competing interests

The authors declare that they have no competing interests.

### Generative AI statement 

AI is not used in any part of writing this manuscript

## Figures and Tables

**Table 1:**
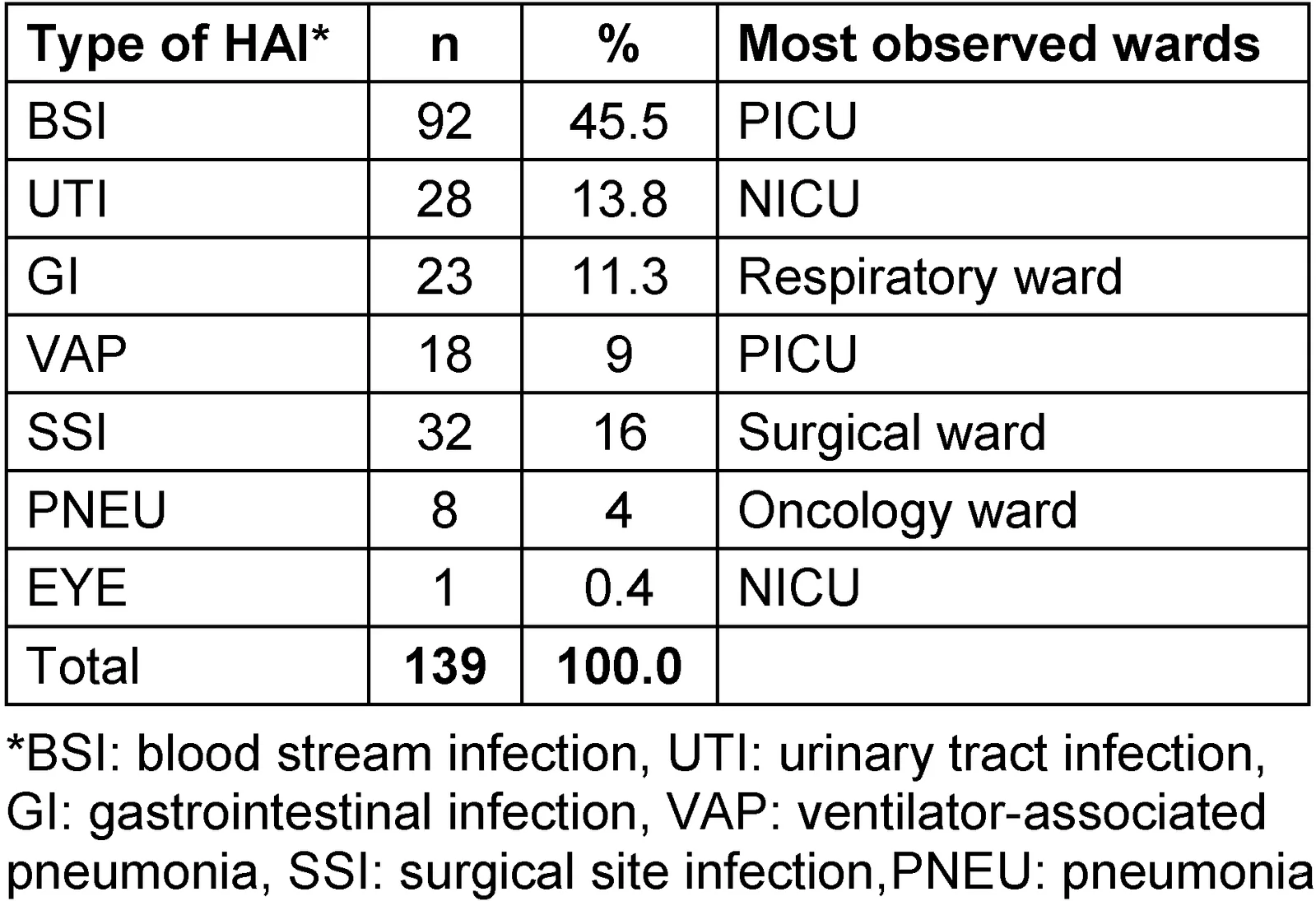
Type of HAIs

**Table 2:**
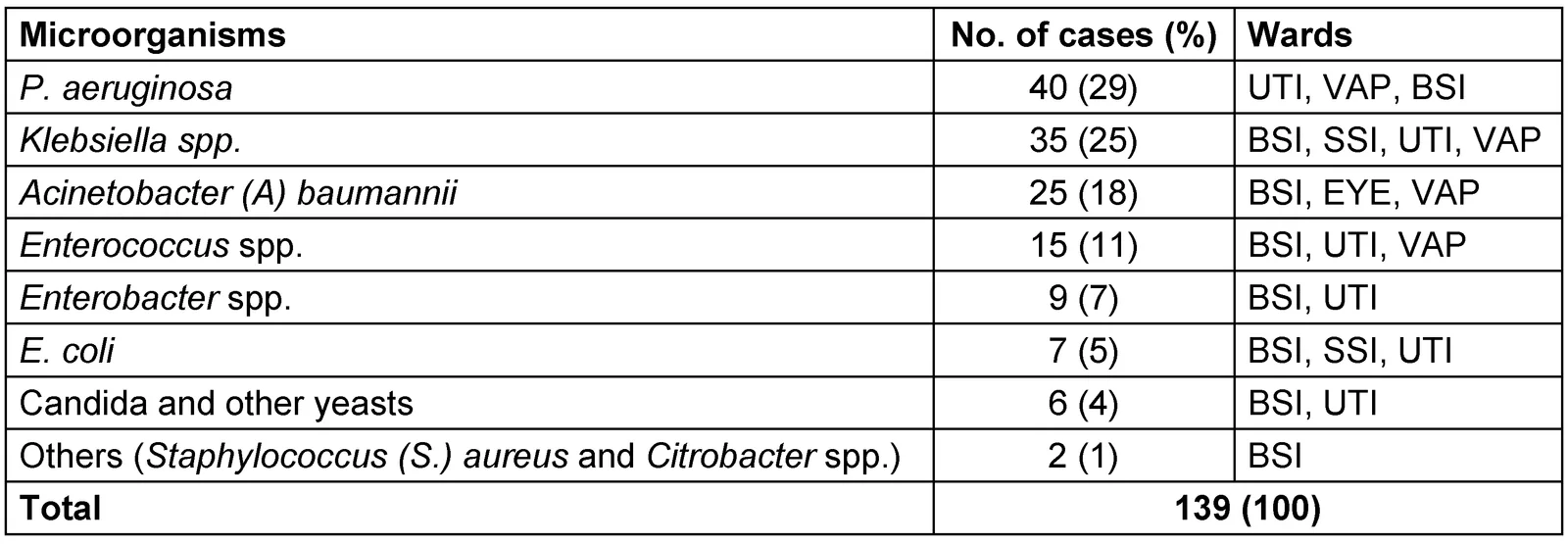
Isolated microorganisms in HAIs

**Table 3:**
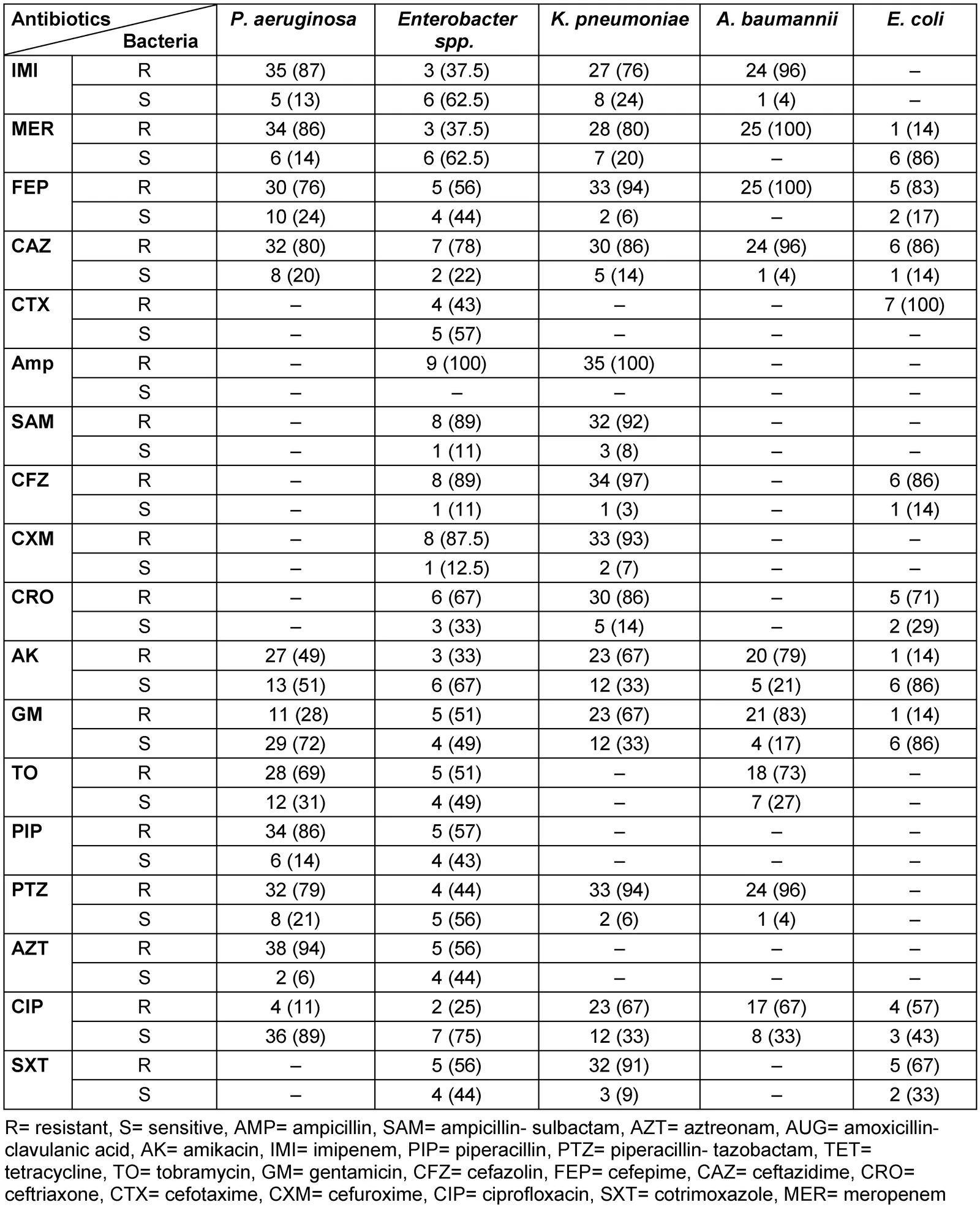
Number/% of antibiotic susceptibility of isolated Gram-negative bacteria in HAIs

**Figure 1:**
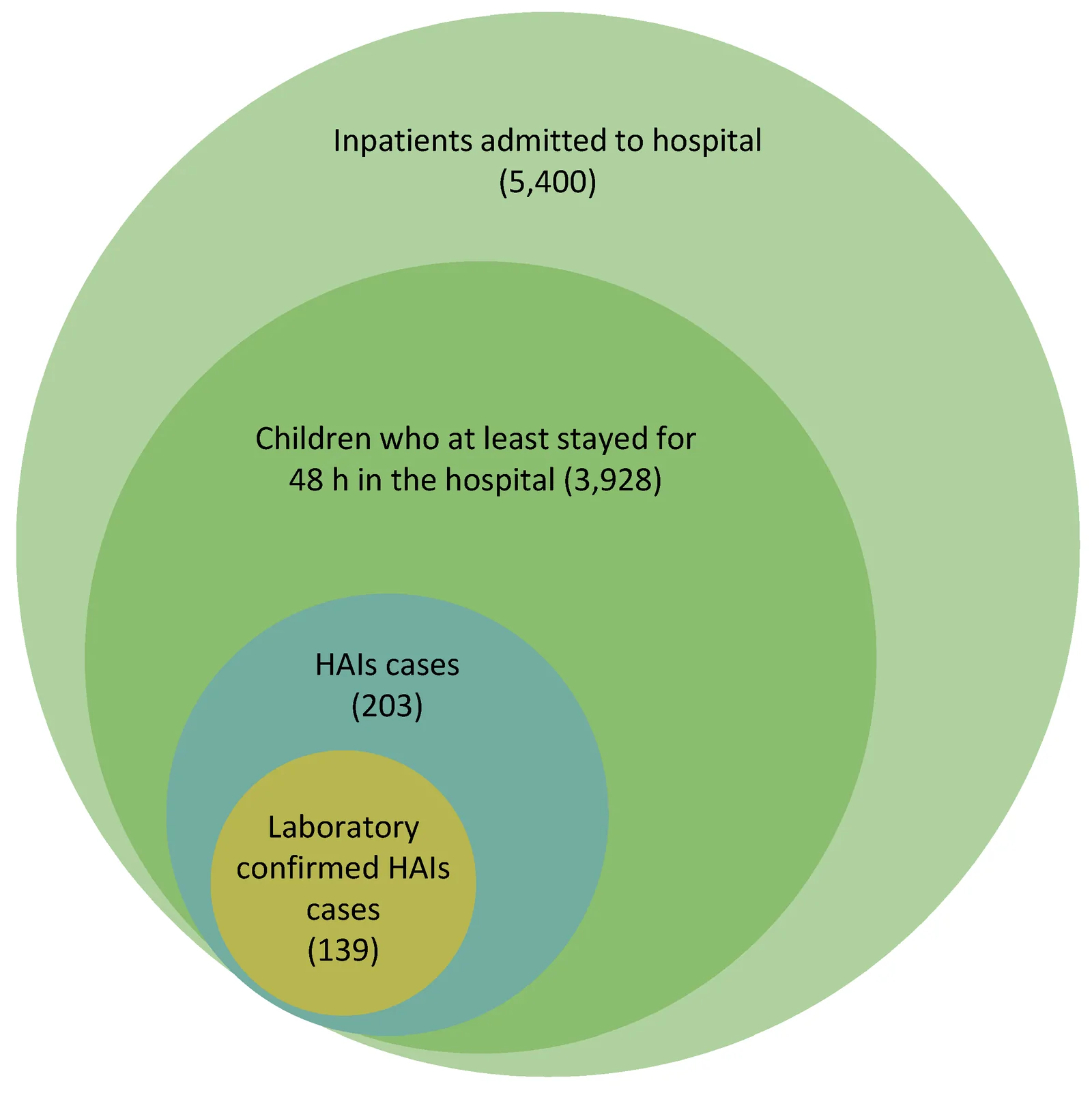
Study population
